# [4′-(3-Pyrid­yl)-2,2′;6′,2′′-terpyridine]dithio­cyanato­copper(II)

**DOI:** 10.1107/S1600536810010147

**Published:** 2010-03-24

**Authors:** Dongcheng Hu, Hua Feng, Changqiu Hu

**Affiliations:** a College of Chemistry & Chemical Engineering, Northwest Normal University, Lanzhou, Gansu 730070, People’s Republic of China

## Abstract

In the title compound, [Cu(NCS)_2_(C_20_H_14_N_4_)], the Cu atom is five-coordinated in a tetra­gonal-pyramidal geometry.

## Related literature

For details of the synthesis, see: Constable & Thompson (1992 ▶). For related structures, see: Feng *et al.* (2006 ▶); Hou *et al.* (2004 ▶).
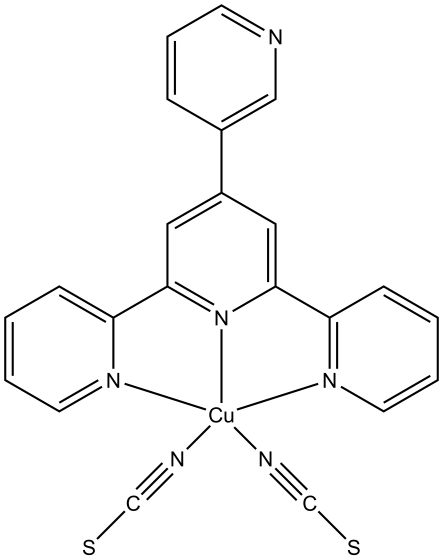


## Experimental

### 

#### Crystal data

[Cu(NCS)_2_(C_20_H_14_N_4_)]
*M* *_r_* = 490.05
Monoclinic, 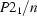

*a* = 8.2171 (6) Å
*b* = 23.012 (2) Å
*c* = 11.2279 (8) Å
β = 99.079 (1)°
*V* = 2096.5 (3) Å^3^
*Z* = 4
Mo *K*α radiation
μ = 1.26 mm^−1^
*T* = 298 K
0.27 × 0.27 × 0.18 mm

#### Data collection

Bruker SMART APEX area-detector diffractometer
Absorption correction: multi-scan (*SADABS*; Bruker, 2002 ▶) *T*_min_ = 0.429, *T*_max_ = 0.805
12626 measured reflections
4781 independent reflections
3371 reflections with *I* > 2σ(*I*)
*R*_int_ = 0.038

#### Refinement

*R*[*F* ^2^ > 2σ(*F* ^2^)] = 0.059
*wR*(*F* ^2^) = 0.160
*S* = 1.07
4781 reflections
280 parameters
H-atom parameters constrained
Δρ_max_ = 0.64 e Å^−3^
Δρ_min_ = −0.44 e Å^−3^

### 

Data collection: *SMART* (Bruker, 2002 ▶); cell refinement: *SAINT* (Bruker, 2002 ▶); data reduction: *SAINT*; program(s) used to solve structure: *SHELXS97* (Sheldrick, 2008 ▶); program(s) used to refine structure: *SHELXL97* (Sheldrick, 2008 ▶); molecular graphics: *ORTEP-3* (Farrugia, 1997 ▶); software used to prepare material for publication: *SHELXL97*.

## Supplementary Material

Crystal structure: contains datablocks I, global. DOI: 10.1107/S1600536810010147/bt5216sup1.cif

Structure factors: contains datablocks I. DOI: 10.1107/S1600536810010147/bt5216Isup2.hkl

Additional supplementary materials:  crystallographic information; 3D view; checkCIF report

## Figures and Tables

**Fig. 1.**
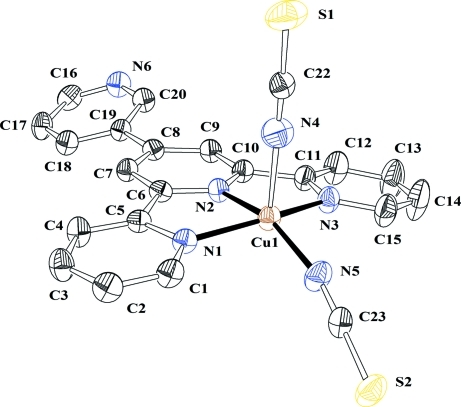
ORTEP-3 plot of the title compound with the atom labeling scheme. Displacement ellipsoids are drawn at the 35% probability level. H atoms are omitted for clarity.

## supplementary crystallographic information

### Comment

The heterocyclic ligand 4'-(3-pyridyl)-2,2':6'2''-terpyridine was synthesized according to literature methods (Constable *et al.*, 1992). As a excellent tridentate chelating ligand, it can furnish  complexes from a large range of metal salts. The structure of the title compound, (I), obtained from copper(I) thiocyanate is shown in Fig. 1. The Cu(II) atom is chelated by a tridentate terpydine in a *cis-cis* configuration and two independent thiocyanate in a five-coordinate environment and shows distorted tetragonal pyramid geometry (Fig. 1). The Cu—N bonds range from 1.931 (3)Å to 2.143 (4)Å are comparable with those reported (Hou *et al.*, 2004). In the monomer, one thiocyanate was in the apical site while the terpyridine and the other one thiocyanate were at the equatorial positions.

### Experimental

The mixture of CuSCN (0.0125 g, 0.1 mmol), 4'-(3-pyridyl)-2,2':6'2''-terpyridine (0.0155 g, 0.05 mmol), acetonnitril (6 ml) were placed and sealed in a 10 ml Teflon-lined stainless steel reactor and heated to 140°C for 72 h, then cooled down to room temperature at a rate of 2°C per 20 min. Green block single crystals suitable for X-ray diffraction were obtained in ca. 60% yield

### Refinement

Hydrogen atoms were placed in calculated positions (C–H 0.93 Å; U(H) = 1.2U_eq_C) and were included in the refinement in the riding model approximation.

### Crystal data

**Table:**

[Cu(NCS)_2_(C_20_H_14_N_4_)]	*F*(000) = 996
*M**_r_* = 490.05	*D*_x_ = 1.553 Mg m^−^^3^
Monoclinic, *P*2_1_/*n*	Mo *K*α radiation, λ = 0.71073 Å
Hall symbol: -P 2yn	Cell parameters from 2079 reflections
*a* = 8.2171 (6) Å	θ = 2.6–21.4°
*b* = 23.012 (2) Å	µ = 1.26 mm^−^^1^
*c* = 11.2279 (8) Å	*T* = 298 K
β = 99.079 (1)°	Block, green
*V* = 2096.5 (3) Å^3^	0.27 × 0.27 × 0.18 mm
*Z* = 4	

### Data collection

**Table:**

Bruker SMART APEX area-detector diffractometer	4781 independent reflections
Radiation source: fine-focus sealed tube	3371 reflections with *I* > 2σ(*I*)
graphite	*R*_int_ = 0.038
φ and ω scan	θ_max_ = 27.5°, θ_min_ = 1.8°
Absorption correction: multi-scan (*SADABS*; Bruker, 2002)	*h* = −10→5
*T*_min_ = 0.429, *T*_max_ = 0.805	*k* = −26→29
12626 measured reflections	*l* = −14→14

### Refinement

**Table:**

Refinement on *F*^2^	Primary atom site location: structure-invariant direct methods
Least-squares matrix: full	Secondary atom site location: difference Fourier map
*R*[*F*^2^ > 2σ(*F*^2^)] = 0.059	Hydrogen site location: inferred from neighbouring sites
*wR*(*F*^2^) = 0.160	H-atom parameters constrained
*S* = 1.07	*w* = 1/[σ^2^(*F*_o_^2^) + (0.0832*P*)^2^] where *P* = (*F*_o_^2^ + 2*F*_c_^2^)/3
4781 reflections	(Δ/σ)_max_ = 0.001
280 parameters	Δρ_max_ = 0.64 e Å^−^^3^
0 restraints	Δρ_min_ = −0.44 e Å^−^^3^

### Fractional atomic coordinates and isotropic or equivalent isotropic displacement parameters (Å^2^)

**Table:**

	*x*	*y*	*z*	*U*_iso_*/*U*_eq_	
Cu1	0.35786 (5)	0.135609 (18)	0.69095 (4)	0.04072 (18)	
S1	0.5487 (2)	0.10524 (7)	0.30631 (12)	0.0930 (5)	
S2	0.69634 (16)	0.05042 (5)	1.01610 (12)	0.0705 (4)	
N1	0.4648 (4)	0.21527 (13)	0.7228 (3)	0.0395 (7)	
N2	0.1730 (3)	0.18662 (12)	0.6333 (3)	0.0376 (7)	
N3	0.1684 (4)	0.07761 (13)	0.6675 (3)	0.0477 (8)	
N4	0.4448 (5)	0.12136 (17)	0.5232 (4)	0.0681 (11)	
N5	0.5115 (4)	0.09198 (15)	0.8049 (3)	0.0567 (9)	
N6	−0.4985 (4)	0.30689 (15)	0.3820 (3)	0.0545 (9)	
C1	0.6201 (4)	0.22617 (17)	0.7760 (4)	0.0459 (9)	
H1	0.6878	0.1950	0.8032	0.055*	
C2	0.6827 (5)	0.28129 (18)	0.7918 (4)	0.0517 (10)	
H2	0.7899	0.2875	0.8306	0.062*	
C3	0.5841 (5)	0.32709 (17)	0.7493 (4)	0.0544 (11)	
H3	0.6247	0.3648	0.7579	0.065*	
C4	0.4239 (5)	0.31726 (16)	0.6935 (4)	0.0473 (10)	
H4	0.3556	0.3481	0.6649	0.057*	
C5	0.3680 (4)	0.26104 (15)	0.6813 (3)	0.0369 (8)	
C6	0.1981 (4)	0.24383 (15)	0.6274 (3)	0.0362 (8)	
C7	0.0730 (4)	0.28084 (15)	0.5800 (3)	0.0394 (8)	
H7	0.0929	0.3204	0.5741	0.047*	
C8	−0.0849 (4)	0.25821 (15)	0.5408 (3)	0.0378 (8)	
C9	−0.1082 (4)	0.19839 (16)	0.5516 (3)	0.0414 (9)	
H9	−0.2116	0.1820	0.5272	0.050*	
C10	0.0237 (4)	0.16374 (15)	0.5987 (3)	0.0396 (8)	
C11	0.0177 (5)	0.10032 (16)	0.6220 (4)	0.0475 (10)	
C12	−0.1211 (6)	0.0675 (2)	0.6069 (5)	0.0726 (15)	
H12	−0.2220	0.0839	0.5749	0.087*	
C13	−0.1112 (7)	0.0091 (2)	0.6397 (6)	0.094 (2)	
H13	−0.2052	−0.0140	0.6326	0.113*	
C14	0.0431 (7)	−0.0137 (2)	0.6833 (6)	0.097 (2)	
H14	0.0545	−0.0529	0.7031	0.116*	
C15	0.1769 (6)	0.02138 (17)	0.6969 (5)	0.0661 (13)	
H15	0.2791	0.0057	0.7280	0.079*	
C16	−0.4894 (5)	0.36367 (19)	0.4057 (4)	0.0554 (11)	
H16	−0.5805	0.3866	0.3774	0.066*	
C17	−0.3527 (5)	0.39026 (18)	0.4697 (4)	0.0550 (11)	
H17	−0.3514	0.4301	0.4835	0.066*	
C18	−0.2186 (5)	0.35648 (16)	0.5128 (4)	0.0482 (10)	
H18	−0.1246	0.3734	0.5557	0.058*	
C19	−0.2235 (4)	0.29699 (15)	0.4923 (3)	0.0374 (8)	
C20	−0.3672 (5)	0.27481 (17)	0.4255 (4)	0.0480 (10)	
H20	−0.3720	0.2351	0.4102	0.058*	
C22	0.4845 (5)	0.11376 (17)	0.4333 (4)	0.0441 (9)	
C23	0.5890 (5)	0.07416 (16)	0.8920 (4)	0.0468 (9)	

### Atomic displacement parameters (Å^2^)

**Table:**

	*U*^11^	*U*^22^	*U*^33^	*U*^12^	*U*^13^	*U*^23^
Cu1	0.0325 (3)	0.0397 (3)	0.0473 (3)	0.00621 (19)	−0.0019 (2)	0.00460 (19)
S1	0.1100 (13)	0.1146 (12)	0.0590 (8)	0.0514 (10)	0.0277 (8)	0.0161 (8)
S2	0.0649 (8)	0.0655 (8)	0.0726 (8)	−0.0015 (6)	−0.0159 (6)	0.0226 (6)
N1	0.0289 (15)	0.0434 (17)	0.0445 (17)	0.0030 (13)	0.0009 (13)	0.0023 (13)
N2	0.0282 (15)	0.0349 (15)	0.0471 (18)	0.0007 (12)	−0.0026 (13)	−0.0006 (13)
N3	0.0423 (18)	0.0389 (17)	0.057 (2)	0.0000 (14)	−0.0059 (16)	0.0046 (15)
N4	0.069 (3)	0.073 (3)	0.064 (3)	0.017 (2)	0.015 (2)	0.001 (2)
N5	0.045 (2)	0.055 (2)	0.065 (2)	0.0071 (17)	−0.0078 (18)	0.0152 (17)
N6	0.0328 (17)	0.061 (2)	0.066 (2)	0.0045 (16)	−0.0062 (16)	0.0049 (17)
C1	0.0250 (18)	0.052 (2)	0.057 (2)	0.0032 (16)	−0.0053 (17)	0.0087 (18)
C2	0.0277 (18)	0.062 (3)	0.062 (3)	−0.0025 (18)	−0.0034 (18)	0.004 (2)
C3	0.039 (2)	0.045 (2)	0.077 (3)	−0.0086 (19)	0.003 (2)	−0.002 (2)
C4	0.0312 (19)	0.040 (2)	0.069 (3)	0.0007 (17)	0.0017 (18)	0.0014 (18)
C5	0.0270 (17)	0.044 (2)	0.0386 (19)	0.0037 (15)	0.0016 (15)	0.0003 (15)
C6	0.0280 (17)	0.0418 (19)	0.0378 (19)	0.0031 (15)	0.0021 (15)	−0.0020 (15)
C7	0.0333 (19)	0.0378 (19)	0.045 (2)	0.0023 (15)	−0.0012 (16)	0.0035 (15)
C8	0.0298 (18)	0.041 (2)	0.041 (2)	0.0018 (15)	−0.0014 (15)	−0.0007 (15)
C9	0.0291 (18)	0.042 (2)	0.049 (2)	−0.0011 (16)	−0.0045 (16)	−0.0009 (16)
C10	0.0327 (19)	0.039 (2)	0.045 (2)	0.0014 (16)	−0.0003 (16)	0.0026 (16)
C11	0.044 (2)	0.039 (2)	0.055 (2)	−0.0036 (17)	−0.0060 (19)	0.0045 (17)
C12	0.048 (3)	0.057 (3)	0.102 (4)	−0.009 (2)	−0.022 (3)	0.015 (3)
C13	0.069 (3)	0.052 (3)	0.146 (5)	−0.022 (3)	−0.029 (4)	0.024 (3)
C14	0.084 (4)	0.047 (3)	0.144 (6)	−0.011 (3)	−0.034 (4)	0.033 (3)
C15	0.061 (3)	0.042 (2)	0.088 (3)	0.003 (2)	−0.012 (3)	0.014 (2)
C16	0.036 (2)	0.063 (3)	0.064 (3)	0.016 (2)	0.000 (2)	0.015 (2)
C17	0.047 (2)	0.041 (2)	0.077 (3)	0.0076 (19)	0.008 (2)	0.007 (2)
C18	0.034 (2)	0.049 (2)	0.057 (2)	−0.0013 (17)	−0.0050 (18)	0.0067 (18)
C19	0.0278 (17)	0.0392 (19)	0.044 (2)	0.0032 (15)	0.0019 (15)	0.0036 (15)
C20	0.035 (2)	0.048 (2)	0.057 (2)	0.0032 (17)	−0.0060 (18)	−0.0001 (18)
C22	0.036 (2)	0.044 (2)	0.050 (2)	0.0082 (17)	0.0011 (18)	0.0021 (18)
C23	0.036 (2)	0.038 (2)	0.065 (3)	−0.0003 (17)	0.0044 (19)	0.0053 (18)

### Geometric parameters (Å, °)

**Table:**

Cu1—N1	2.040 (3)	C5—C6	1.485 (4)
Cu1—N2	1.948 (3)	C6—C7	1.375 (5)
Cu1—N3	2.036 (3)	C7—C8	1.403 (5)
Cu1—N4	2.143 (4)	C7—H7	0.9300
Cu1—N5	1.931 (3)	C8—C9	1.398 (5)
S1—C22	1.609 (5)	C8—C19	1.482 (5)
S2—C23	1.622 (4)	C9—C10	1.382 (5)
N1—C1	1.344 (4)	C9—H9	0.9300
N1—C5	1.357 (4)	C10—C11	1.485 (5)
N2—C10	1.335 (4)	C11—C12	1.356 (6)
N2—C6	1.336 (4)	C12—C13	1.395 (6)
N3—C15	1.334 (5)	C12—H12	0.9300
N3—C11	1.366 (5)	C13—C14	1.388 (7)
N4—C22	1.122 (5)	C13—H13	0.9300
N5—C23	1.154 (5)	C14—C15	1.353 (6)
N6—C16	1.333 (5)	C14—H14	0.9300
N6—C20	1.334 (5)	C15—H15	0.9300
C1—C2	1.370 (5)	C16—C17	1.378 (6)
C1—H1	0.9300	C16—H16	0.9300
C2—C3	1.369 (5)	C17—C18	1.373 (5)
C2—H2	0.9300	C17—H17	0.9300
C3—C4	1.384 (5)	C18—C19	1.388 (5)
C3—H3	0.9300	C18—H18	0.9300
C4—C5	1.373 (5)	C19—C20	1.392 (5)
C4—H4	0.9300	C20—H20	0.9300
			
N1—Cu1—N2	79.0 (1)	C8—C7—H7	120.4
N1—Cu1—N3	155.7 (1)	C9—C8—C7	118.1 (3)
N1—Cu1—N4	95.6 (1)	C9—C8—C19	121.2 (3)
N1—Cu1—N5	97.6 (1)	C7—C8—C19	120.8 (3)
N2—Cu1—N3	79.1 (1)	C10—C9—C8	119.5 (3)
N2—Cu1—N4	98.7 (1)	C10—C9—H9	120.3
N2—Cu1—N5	158.0 (2)	C8—C9—H9	120.3
N3—Cu1—N4	98.1 (2)	N2—C10—C9	120.9 (3)
N3—Cu1—N5	98.7 (1)	N2—C10—C11	113.0 (3)
N4—Cu1—N5	103.2 (2)	C9—C10—C11	126.1 (3)
C1—N1—C5	118.1 (3)	C12—C11—N3	121.9 (4)
C1—N1—Cu1	126.7 (3)	C12—C11—C10	125.1 (4)
C5—N1—Cu1	115.1 (2)	N3—C11—C10	112.9 (3)
C10—N2—C6	121.1 (3)	C11—C12—C13	119.4 (4)
C10—N2—Cu1	119.5 (2)	C11—C12—H12	120.3
C6—N2—Cu1	119.5 (2)	C13—C12—H12	120.3
C15—N3—C11	118.2 (3)	C14—C13—C12	118.0 (5)
C15—N3—Cu1	126.5 (3)	C14—C13—H13	121.0
C11—N3—Cu1	115.3 (2)	C12—C13—H13	121.0
C22—N4—Cu1	177.4 (4)	C15—C14—C13	119.7 (4)
C23—N5—Cu1	164.0 (4)	C15—C14—H14	120.2
C16—N6—C20	116.9 (3)	C13—C14—H14	120.2
N1—C1—C2	122.8 (3)	N3—C15—C14	122.7 (4)
N1—C1—H1	118.6	N3—C15—H15	118.6
C2—C1—H1	118.6	C14—C15—H15	118.6
C3—C2—C1	118.6 (3)	N6—C16—C17	123.7 (4)
C3—C2—H2	120.7	N6—C16—H16	118.2
C1—C2—H2	120.7	C17—C16—H16	118.2
C2—C3—C4	120.0 (4)	C18—C17—C16	118.4 (4)
C2—C3—H3	120.0	C18—C17—H17	120.8
C4—C3—H3	120.0	C16—C17—H17	120.8
C5—C4—C3	118.6 (4)	C17—C18—C19	120.0 (4)
C5—C4—H4	120.7	C17—C18—H18	120.0
C3—C4—H4	120.7	C19—C18—H18	120.0
N1—C5—C4	121.9 (3)	C18—C19—C20	116.8 (3)
N1—C5—C6	113.3 (3)	C18—C19—C8	122.2 (3)
C4—C5—C6	124.8 (3)	C20—C19—C8	121.0 (3)
N2—C6—C7	121.2 (3)	N6—C20—C19	124.2 (4)
N2—C6—C5	112.6 (3)	N6—C20—H20	117.9
C7—C6—C5	126.1 (3)	C19—C20—H20	117.9
C6—C7—C8	119.3 (3)	N4—C22—S1	177.1 (5)
C6—C7—H7	120.4	N5—C23—S2	178.6 (4)
			
N5—Cu1—N1—C1	18.8 (3)	N1—C5—C6—N2	1.7 (4)
N2—Cu1—N1—C1	176.8 (3)	C4—C5—C6—N2	−176.0 (3)
N3—Cu1—N1—C1	150.6 (3)	N1—C5—C6—C7	178.9 (3)
N4—Cu1—N1—C1	−85.4 (3)	C4—C5—C6—C7	1.2 (6)
N5—Cu1—N1—C5	−163.6 (3)	N2—C6—C7—C8	2.0 (5)
N2—Cu1—N1—C5	−5.6 (2)	C5—C6—C7—C8	−175.0 (3)
N3—Cu1—N1—C5	−31.8 (4)	C6—C7—C8—C9	−0.1 (5)
N4—Cu1—N1—C5	92.2 (3)	C6—C7—C8—C19	178.4 (3)
N5—Cu1—N2—C10	−91.2 (4)	C7—C8—C9—C10	−0.7 (5)
N3—Cu1—N2—C10	−4.8 (3)	C19—C8—C9—C10	−179.1 (3)
N1—Cu1—N2—C10	−174.1 (3)	C6—N2—C10—C9	2.3 (5)
N4—Cu1—N2—C10	91.8 (3)	Cu1—N2—C10—C9	−176.6 (3)
N5—Cu1—N2—C6	89.9 (4)	C6—N2—C10—C11	−175.0 (3)
N3—Cu1—N2—C6	176.3 (3)	Cu1—N2—C10—C11	6.0 (4)
N1—Cu1—N2—C6	6.9 (3)	C8—C9—C10—N2	−0.4 (6)
N4—Cu1—N2—C6	−87.1 (3)	C8—C9—C10—C11	176.6 (4)
N5—Cu1—N3—C15	−16.7 (4)	C15—N3—C11—C12	0.3 (7)
N2—Cu1—N3—C15	−174.5 (4)	Cu1—N3—C11—C12	−176.8 (4)
N1—Cu1—N3—C15	−148.3 (4)	C15—N3—C11—C10	177.2 (4)
N4—Cu1—N3—C15	88.1 (4)	Cu1—N3—C11—C10	0.0 (4)
N5—Cu1—N3—C11	160.2 (3)	N2—C10—C11—C12	173.0 (4)
N2—Cu1—N3—C11	2.4 (3)	C9—C10—C11—C12	−4.1 (7)
N1—Cu1—N3—C11	28.5 (5)	N2—C10—C11—N3	−3.7 (5)
N4—Cu1—N3—C11	−95.0 (3)	C9—C10—C11—N3	179.1 (4)
N2—Cu1—N5—C23	−15.6 (15)	N3—C11—C12—C13	0.7 (8)
N3—Cu1—N5—C23	−98.2 (12)	C10—C11—C12—C13	−175.7 (5)
N1—Cu1—N5—C23	63.7 (12)	C11—C12—C13—C14	−2.1 (10)
N4—Cu1—N5—C23	161.3 (12)	C12—C13—C14—C15	2.5 (11)
C5—N1—C1—C2	1.3 (6)	C11—N3—C15—C14	0.1 (8)
Cu1—N1—C1—C2	178.8 (3)	Cu1—N3—C15—C14	176.9 (5)
N1—C1—C2—C3	−1.5 (7)	C13—C14—C15—N3	−1.5 (10)
C1—C2—C3—C4	1.1 (7)	C20—N6—C16—C17	1.2 (7)
C2—C3—C4—C5	−0.5 (7)	N6—C16—C17—C18	−0.7 (7)
C1—N1—C5—C4	−0.7 (5)	C16—C17—C18—C19	−0.5 (6)
Cu1—N1—C5—C4	−178.5 (3)	C17—C18—C19—C20	1.2 (6)
C1—N1—C5—C6	−178.5 (3)	C17—C18—C19—C8	−177.6 (4)
Cu1—N1—C5—C6	3.7 (4)	C9—C8—C19—C18	160.4 (4)
C3—C4—C5—N1	0.4 (6)	C7—C8—C19—C18	−18.0 (6)
C3—C4—C5—C6	177.9 (4)	C9—C8—C19—C20	−18.2 (6)
C10—N2—C6—C7	−3.1 (5)	C7—C8—C19—C20	163.3 (4)
Cu1—N2—C6—C7	175.8 (3)	C16—N6—C20—C19	−0.4 (6)
C10—N2—C6—C5	174.2 (3)	C18—C19—C20—N6	−0.7 (6)
Cu1—N2—C6—C5	−6.8 (4)	C8—C19—C20—N6	178.0 (4)
